# Herpesviruses in migrating procellariforms, northeastern Brazil

**DOI:** 10.1007/s11259-024-10434-9

**Published:** 2024-06-18

**Authors:** Carlos Sacristán, Aricia Duarte-Benvenuto, Pedro Enrique Navas-Suárez, Roberta Zamana-Ramblas, Laura Baes, Barbara Sophia Codeas, Larissa Pavanelli, Joana Ikeda, José Luiz Catão-Dias, Ana Carolina Ewbank

**Affiliations:** 1 Centro de Investigación en Sanidad Animal (CISA-INIA), CSIC, Valdeolmos, Madrid Spain; 2 Laboratory of Wildlife Comparative Pathology, School of Veterinary Medicine and Animal Science, University of São Paulo, São Paulo, SP Brazil; 3 Laboratório de Ecologia de Interações, Departamento de Ecologia E Biologia Evolutiva, Universidade Federal de São Carlos - UFSCar, São Carlos, SP Brazil; 4 Instituto Mamíferos Aquáticos, Salvador, BA Brazil

**Keywords:** Virology, Procellariidae, Seabirds, South America

## Abstract

Seabirds are one of the most threatened avian groups. Viruses, including herpesvirus, represent considerable threats to marine avifauna. Herein, our goal was to survey herpesvirus in Procellariiformes that stranded in Brazil between June and July 2021. We analyzed 12 Cory's shearwaters (*Calonectris borealis*), two Great Shearwaters (*Ardenna gravis*, syn. *Puffinus gravis*) and one Yellow-nosed Albatross (*Thalassarche chlororynchos*) found in an unusual mortality event in Bahía state, northeastern Brazil. After necropsy, selected tissue samples were tested for herpesvirus using a broad-range nested PCR. Overall, 20% (3/15) of the birds were herpesvirus-positive, i.e., two Cory's Shearwaters and one Great Shearwater. One alphaherpesvirus sequence type was identified in each shearwater species, classified into the genus *Mardivirus*. This study describes two likely novel herpesviruses in shearwaters, contributing to the currently very scarce data regarding infectious agents in Procellariiformes. Further studies are necessary to evaluate the presence and characteristics of herpesvirus in Procellariiformes, and the presence (or not) of related disease in order to understand the epidemiology of this infectious agent and eventually contribute to the conservation of this endangered seabird group.

## Introduction

Seabirds are among the most threatened avian groups, also considered good indicators of marine ecosystems health (Croxall et al. 2012). Epidemiological surveillance provides crucial information regarding pathogens’ hosts and geographic distribution, shedding light into the ecology, epidemiology and potential impact of diseases, a crucial strategy in wildlife conservation. Viruses, including herpesvirus, represent considerable threats to marine avifauna and birds undergoing rehabilitation, as previously reported in mortality outbreaks worldwide (Niemeyer et al. 2017; Sebastiano et al. 2020; Ewbank et al. 2023).

Herpesviruses (order *Herpesvirales*) are linear, enveloped, double-stranded DNA viruses able to infect vertebrates and some invertebrate species (Davison et al. 2009). The members of the family *Orthoherpesviridae*, namely the subfamilies *Alpha-*, *Beta-* and *Gammaherpesvirinae*, are thought to have coevolved with their host species, generally showing low pathogenicity and establishing latent infections in their natural hosts (Davison et al. 2009). Less commonly, immune challenges (e.g., concomitant disease, captivity or intoxication by chemical pollutants) may trigger viral reactivation, leading to disease and even death of the natural hosts (Kaleta and Docherty 2007; Niemeyer et al. 2017). Additionally, cross-species infection (“spillovers”) may cause severe disease and high mortality, even leading to severe outbreaks (Kaleta and Docherty 2007).

To this date, alphaherpesvirus within the genera *Iltovirus* and *Mardivirus* have been described in a variety of avian orders (Davison et al. 2009; Niemeyer et al. 2017; de Francisco et al. 2023; Ewbank et al. 2023). In seabirds, several alphaherpesviruses have been described in Charadriiformes, Gaviiformes, Procellariiformes, Phaethontiformes, Sphenisciformes, and Suliformes (Quesada et al. 2011; Niemeyer et al. 2017; Verdugo et al. 2019; Sebastiano et al. 2020); however, little is known about herpesviruses in Procellariiformes, limited to the description of a herpesvirus in a Yellow-nosed Albatross (*Thalassarche chlororynchos*) (Niemeyer et al. 2017). Stranding events involving pelagic seabirds provide an opportunity to assess population threats, infectious agents and related stressors. Thus, our goal was to molecularly survey and characterize herpesviruses in migrating Procellariiformes stranded alive in northeastern Brazilian coast and admitted into a rehabilitation center.

## Material and methods

### Samples

Overall, 12 Cory's Shearwaters (*Calonectris borealis*, fam. Procellariidae; 5 males, 6 females, and 1 undetermined; 5 adults, 5 juveniles, and 2 undetermined), two Great Shearwaters (*Ardenna gravis*, syn. *Puffinus gravis*, family Procellariidae; 1 adult male and 1 adult of unknown age), and one Atlantic Yellow-nosed Albatross (fam. Diomedeidae; juvenile female) were found debilitated during an unusual mortality event in a coastal strip of Bahía state (13º0′36.216″ S -38º31′3.468″ W to 12º21′39.06″ S -37º51′43.164″ W), northeastern Brazil, between June and July 2021.

Rescue and postmortem examinations were performed by the Instituto Mamíferos Aquáticos (IMA), a rehabilitation center in the same state. All animals died, either during transport (n = 2) or while undergoing rehabilitation (n = 13), being necropsied following standard procedures (Hocken 2002). The body condition was classified into good, moderate, poor, or cachectic, based on pectoral muscle development and presence (or absence) of internal and subcutaneous fat deposits. Age class and sex were determined based on plumage pattern and upon visualization of the gonads. Tissue samples (i.e., lungs, brain, liver, spleen, and kidneys) were collected and fixed in 10% buffered formalin and frozen at -20/-80 °C until processed.

### Molecular techniques

Total DNA of selected frozen tissue samples (brain, lung and kidney) was extracted using the DNeasy Blood and Tissue kit, according to the manufacturer's guidelines. No swabs were available. Subsequently, DNA was tested with a broad-range nested PCR protocol for herpesviruses, with an annealing temperature of 46 ºC for both PCRs, able to amplify a fragment of approximately 215–315 bp of the DNA polymerase gene of different alpha, beta- and gammaherpesviruses (VanDevanter et al. 1996). In herpesvirus-positive animals, other available frozen tissue samples (spleen and liver) were also tested following the same methodology described above. A previously confirmed cetacean gammaherpesvirus positive sample was used as positive and DPEC water was used as no template control. All amplicons of the expected size were purified with ExoSAP-IT and directionally sequenced. The obtained consensus sequences were analyzed for further phylogenetic classification using Mega 7.0. Subsequently, a DNA polymerase amino acid maximum likelihood phylogram was constructed including the obtained sequences, representative sequences of the genera *Mardivirus* and *Iltovirus,* and *Human gammaherpesvirus 8* as an outgroup, using Mega 7.0.

### Histopathology

Light microscopy histopathological evaluation was performed in the HV-PCR-positive individuals, on formalin-fixed paraffin-embedded tissues sectioned at 5 μm and stained with hematoxylin and eosin.

## Results

### Molecular findings

Three animals were herpesvirus-positive; two tested Cory's Shearwaters (IMA4473 and IMA4486; 2/12; 16.7%), and one Great Shearwater (IMA4476; 1/2; 50%). The Yellow-nosed Albatross was herpesvirus-negative (Table 1).

**Table 1:** Species, age, sex, gross and histopathologic findings, and comments regarding the herpesvirus-PCR-positive Cory's Shearwater (*Calonectris borealis*) and Great Shearwater (*Ardenna gravis*) described in this study

ID#	Species	Age	Sex	Gross Findings	Histopathology	Comments
4473	*Calonectris borealis*	J	M	Poor body condition, mild jugular vein distention, 2 cm necrotic tear in the proventriculum, presence of partially digested food in the ventriculum. Immature testicles.	Several foci of moderate perivascular pulmonary edema. No histopathological changes were observed in heart, kidney and proventriculum tissues. Postmortem changes compromised the histopathological evaluation of intestines, pancreas, and brain. Inconclusive histopathological examination.	Died while undergoing treatment, following a surgical procedure to remove a fish hook located in proventriculum.
4476	*Ardenna gravis*	A	M	Fair body condition, hydropericardium, focal proventricular serosal congestion, presence of partially digested food and debris in the ventriculum, celomic exudate.	Moderate to discrete multiple foci of perivascular pulmonary edema; moderate multifocal mononuclear (lymphocytic) adrenalitis; moderate multifocal mixed celomitis. No histopathological changes were observed in heart or kidneys. Severe post mortem autolysis compromised the histopathological evaluation of brain, duodenum, liver, adrenal gland, intestines, tongue, pancreas and proventriculum. The observed intestinal inflammatory process and presence of exudate in the celomic cavity could indicate intestinal perforation. No signs of changes related to sepsis or pathogen presence (i.e., bacteria, metazoans or protozoans) were observed.	Died while undergoing treatment. Considerable amount of debris in the stomach. The observed intestinal inflammatory process and presence of exudate in the celomic cavity suggested intestinal perforation. No signs of changes related to sepsis or pathogen presence (i.e., bacteria, metazoans or protozoans) were observed.
4486	*Calonectris borealis*	J	M	Bilateral blepharitis with caseous exudate, mild jugular vein distention, hydropericardium, mild pulmonary edema, pulmonary and renal congestion, presence of partially digested food and sediment (grit) in the ventriculum, presence of celomic exudate, enlarged adrenal glands, and immature testicles.	No histopathological changes were observed in heart or lung. Postmortem changes compromised the histopathological evaluation of brain, liver, tongue and pancreas. The histopathological examination of this individual was not conclusive to determine the causa mortis.	The gross and histopathological examinations of this individual were not conclusive to determine the causa mortis.

*A* adult, *J* juvenile, *M* male

In the Cory's Shearwaters, case IMA4473 was herpesvirus-positive in the spleen and the kidney, and negative in brain, lung and liver; while IMA4486 was only herpesvirus-positive in the lung and brain, and negative in spleen, liver and kidney. Both Cory's Shearwater herpesviral sequences were identical between them, and presented the highest nucleotide (68.9%) and amino acid identities (67.8%) to different Columbid alphaherpesvirus 1 sequences (e.g., GenBank accession nº NC_034266, MW625939, MW625940, detected in Rock Pigeons (*Columba livia*) in China in 2013, and in Slovenia in 2019).

**Fig. 1:**
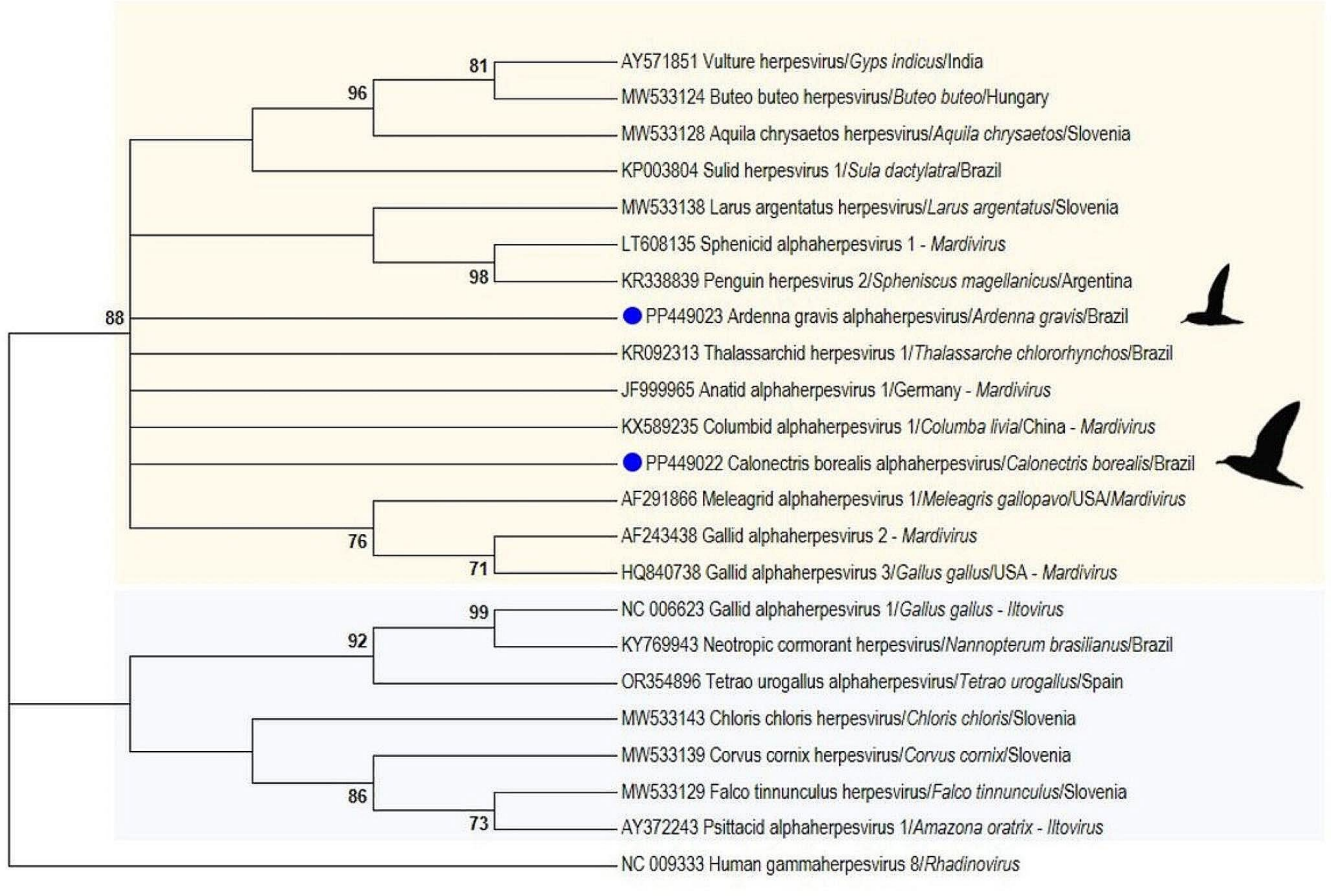
Maximum likelihood phylogram of the alignment of the herpesviral DNA polymerase sequences (1) obtained in this study (blue dots), and (2) previously detected in birds, including those classified into the genus *Mardivirus* (yellow square) and *Iltovirus* (blue square). *Human gammaherpesvirus 8* was selected as outgroup. The phylogram was based on the gamma distributed Le and Gascuel model with Invariant sites and Gamma distribution (LG + I + G). The reliability of the phylogram was tested by a 1000 bootstrap analysis. Bootstrap values lower than 70 were omitted

Regarding the Great Shearwater (IMA4476), lung, spleen, liver and kidney samples were herpesvirus-positive, while the brain was negative. The same herpesviral consensus sequence was obtained in all of this individual’s positive samples, with the highest nucleotide identity (63.5%) to an alphaherpesvirus identified in a Neotropical river otter (*Lontra longicaudis*, GenBank accession nº OQ980217) in Brazil in 2017—although our nucleotide sequence is slightly longer (212 vs. 178), followed by a herpesvirus (60.3%) identified in Common Buzzards (*Buteo buteo*, GenBank accession nº MW533125-MW533127) in Slovenia, in 2018. The highest amino acid (71.6%) identity was to Equid alphaherpesvirus 1 sequences (GenBank accession nº KP689589, KX101098) detected in African elephants (*Loxodonta africana*) in Kenya in 2015 and in a plain zebra (*Equus quagga boehmi*) in Germany in 2014, followed by alphaherpesvirus sequences (67.1%) found in Ural Owls (*Strix uralensis*, GenBank accession nº MH084654, MW315868) and Long-eared Owl (*Asio otus*, GenBank accession nº MH084655, MW533133) of Slovenia identified between 1995 and 2019, and by a Thalassarchid herpesvirus 1 (64.3%) sequence (GenBank accession nº KR092313), detected in the oral swab of an Atlantic Yellow-Nosed Albatross (*Thalassarche chlororhynchos*) in Brazil, in 2013. The sequences obtained in this study clustered within herpesviral sequences of genus *Mardivirus* (Fig. 1).

### Gross and histopathological findings

Overall, tissue samples of herpesvirus-positive individuals were compromised due to advanced autolysis. Nevertheless, in two of the positive birds (Cory's Shearwater 4473 and the herpesvirus-positive Great Shearwater) pulmonary edema was observed. Additionally, mononuclear adrenalitis and moderate mixed celomitis were present in the Great Shearwater (Table 1).

## Discussion

Herein we found two likely novel alphaherpesvirus sequence types, reported for the first time in Cory’s and Great Shearwaters, and in the family Procellariidae. To the authors’ knowledge, these are the second and third descriptions of herpesviruses in Procellariiformes worldwide, following the report of Thalassarchid herpesvirus 1 in one of the 12 Yellow-nosed Albatross under rehabilitation in Rio Grande do Sul State, southern Brazil (Niemeyer et al. 2017). Procellariiformes is a large avian group comprised of long-lived, pelagic species that spend most of their life cycle at sea (Stidworthy and Denk 2018). Due to these particular life history traits, procellariforms are amongst the most endangered threatened taxonomic groups (Croxall et al. 2012), considered highly sensitive to anthropogenic activities (e.g., fisheries interaction, habitat degradation, climate change, invasive and non-native species) (Tavares et al. 2019; Rodríguez et al. 2022). Therefore, data on the infectious agents affecting Procellariiformes is pivotal for the conservation of this group.

The obtained sequence types are highly divergent when compared to the closest from GenBank/DDBJ/EMBL database, with more than 30% of difference. This fact, along with their detection in novel host species, support their classification as novel herpesviral sequences. Nevertheless, further studies detecting more Cory’s and the Great Shearwater individuals infected with these likely novel herpesviruses are necessary in order to establish if these birds are their natural hosts. According to our phylogenetic tree, both our sequence types are classified within the genus *Mardivirus*, which includes relevant avian herpesviruses, such as *Gallid alphaherpesvirus 2* (Marek’s disease) (Davison et al. 2009).

In our study, no associated lesions were observed in any of the herpesvirus-positive individuals, although the advanced autolysis precluded a detailed histopathological examination in some tissue samples. Herpesviruses generally show low virulence in their natural host, which could be the case here (Davison et al. 2009). The herpesvirus-positive species -Cory’s Shearwater and the Great Shearwater- breed, respectively, in Portugal and Canary Islands (Spain), and in the Tristan da Cunha archipelago (United Kingdom) and Malvinas/Falkland Islands, spending their non-breeding season in the Atlantic (Birdlife International 2023a, b). We hypothesize that the stress associated with the long migration of these species from their breeding territories to Brazil likely triggered the reactivation of the detected herpesviruses (Niemeyer et al. 2017), which were detected in several tissue samples.

Large interannual coastal strandings of seabirds (alive and/or dead) have been reported worldwide (Bugoni et al. 2007; Haman et al. 2023; Simeone et al. 2021). The main causes of such events include pollution, weather events (storms and hurricanes, El Niño Southern Oscillation (ENSO), bycatch, starvation (Tavares et al. 2019), and juvenile inexperience (Riotte-Lambert and Weimerskirch 2013). Unfortunately, the causes of the stranding event studied herein were not clear; there were no abnormal or extreme associated weather conditions, the mixed presence of adults and juveniles does not indicate inexperience, and the advanced tissue autolysis prevented a more informative histopathological evaluation.

In summary, this study contributes to the currently very scarce data regarding infectious agents in Procellariiformes by describing two likely novel herpesvirus species in shearwaters. Further studies are necessary to evaluate the presence and characteristics of herpesvirus in Procellariiformes and the presence (or not) of related disease in order to understand the epidemiology of this infectious agent and contribute to the conservation of this endangered seabird group.

## Data Availability

All data generated or analyzed during this study are included in this published article [and its supplementary information files]. The alphaherpesvirus sequences obtained in Cory’s and Great Shearwaters were deposited in GenBank under accession numbers PP449022 and PP449023, respectively.
